# Complex Case Management of Mycobacterium Tuberculosis Extrapulmonary Manifestation to the Right Sacroiliac Joint 

**DOI:** 10.7759/cureus.33789

**Published:** 2023-01-15

**Authors:** Xin Ran Li, Morgan Lucero, Abrahim N Razzak, Pinky Jha, Corrado Ugolini

**Affiliations:** 1 School of Medicine, Medical College of Wisconsin, Milwaukee, USA; 2 Department of Internal Medicine, Medical College of Wisconsin, Milwaukee, USA

**Keywords:** medication interaction, anti-tuberculosis therapy, socioeconomic determinants, sacroiliac infection, mycobacterium tuberculosis, extrapulmonary tuberculosis

## Abstract

While Mycobacterium tuberculosis is a common bacterial pathogen that infects the respiratory system, especially in endemic regions, it may uncommonly manifest in other organ systems, such as the nervous, gastrointestinal, or musculoskeletal systems. Sacroiliac joint infections are rare, and only 1%-5% of all infections are tuberculous in nature. Given nonspecific inflammatory signs in both laboratory and radiologic examinations, early identification of the causative agent can be difficult.

In this report, we present the case of a 29-year-old Eritrean woman who presented with an uncommon extrapulmonary tuberculosis manifestation of the right sacroiliac joint. The patient reported pain for two years before a formal diagnosis with multiple computed tomography scans demonstrated fluid collections about her right hip and thigh. The patient's medical history of developmental delay, psychosis, outdated medication documentation, non-therapeutic use of numerous psychiatric medications contraindicated for traditional anti-tubercular therapy, and socioeconomic history of a lack of social support and treatment arrangements with the patient's caregiver all complicated the treatment course.

Given the rise in tuberculosis cases worldwide and vulnerability factors in patients with mental illnesses such as poverty, homelessness, diabetes, and HIV infection that can predispose patients to tuberculosis infections, early diagnosis and treatment are essential to reduce long-term consequences and improve clinical outcomes. Further research in the development of new tuberculosis treatment plans is essential to addressing an equitable treatment course alongside fighting against the recent rise in drug-resistant tuberculosis.

## Introduction

Infections of the sacroiliac (SI) joint are rare, representing approximately 1-2% of all cases of septic arthritis in recent literature [1], and, as such, diagnoses for infective sacroiliitis (ISI) are delayed. Clinical presentations for ISI are often vague and most frequently accompanied by unilateral lumbogluteal pain with varying degrees of severity and character. They may also present with other non-specific symptoms that mimic pathologies, such as extra-pelvic abscesses, sciatica, or septic arthritis of the hip. Laboratory findings may include increased levels of C-reactive protein (CRP), erythrocyte sedimentation rate (ESR), and white blood cell count (WBC), and radiographic findings are often normal in the early stages of infection [2-5]. Identification of the causative agent of ISI and the treatment course is complex, often causing far-reaching consequences for the patient. In this report, we present the complex treatment case of an extrapulmonary manifestation of Mycobacterium tuberculosis (MTB) in the patient’s right hip.

## Case presentation

A 29-year-old woman, accompanied by her guardian, with a medical history of developmental delay and gastroesophageal reflux disease, presented to the emergency department (ED) for worsening right hip pain. Notably, she emigrated from Eritrea around ten years before this ED visit.

**Figure 1:**
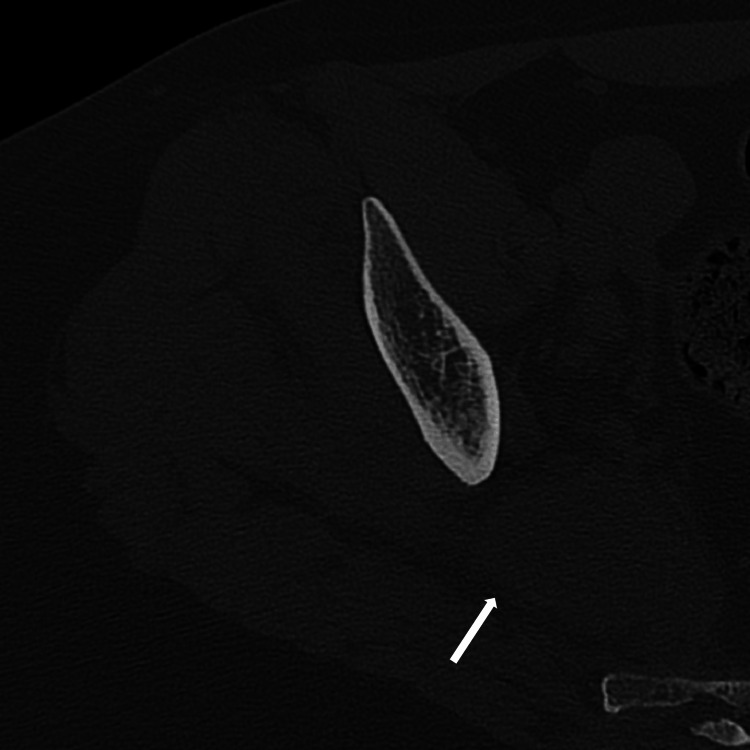
A CT of the right hip taken at urgent care demonstrates abnormal fluid collection in the right piriformis.

Two years prior to this ED visit, the patient's right SI joint pain was evaluated and admitted. When the patient initially presented, she had a six-week history of worsening right hip and buttock pain, which was concerning for a septic joint. The patient was examined and found to have a negative chest x-ray, an elevated erythrocyte sedimentation rate (ESR) of 44 mm/hr (reference range: 0-20 mm/hr), and a C-reactive protein (CRP) of 1.90 mg/dL (reference range: 0-50 mg/dL). Joint aspiration resulted in 1 cc of pus. However, blood cultures (Gram staining) were negative and did not grow any organisms. As such, the patient was discharged after four weeks of intravenous vancomycin and ceftriaxone. She still had chronic SI joint pain, which she managed with morphine or oxycodone. The patient was then seen by urgent care five months ago, where computed tomography (CT) of the right hip showed an abnormal fluid collection in the right piriformis extending to the right acetabulum and destruction of the SI joint (Figure 1). For that visit, she was recommended Tylenol for the pain and was referred to outpatient orthopedics; however, the patient did not follow up with orthopedics.

**Figure 2:**
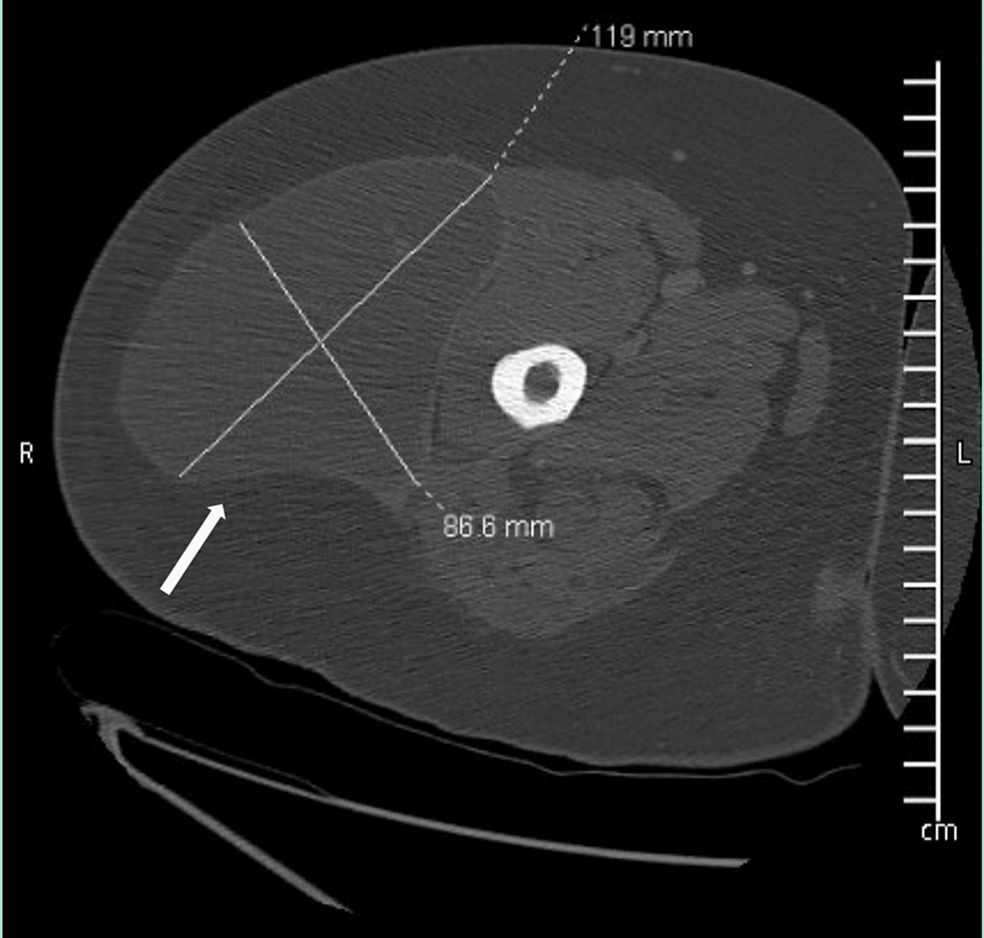
A CT of the right femur with contrast shows rim-enhancing fluid collection measuring 119 x 86.6 mm along the lateral aspect of the thigh.

On the physical exam, the patient appeared uncomfortable from pain but was alert, oriented, and afebrile. The patient spoke slowly in broken English and had a childlike demeanor. On musculoskeletal examination, her right hip demonstrated swelling and tenderness from the lower buttock to the mid-thigh with a normal range of motion (ROM). She was able to bear weight, ambulate, and show normal sensory and motor exam findings. Labs were significant for low hemoglobin (9.7 g/dL) with a normal WBC count. A CT scan of her right hip and femur with contrast showed numerous rim-enhancing fluid collections about the right hip and thigh, with the largest rim-enhancing fluid collection measuring 11.9 x 8.7 x 22.7 cm along the lateral aspect of the thigh (Figure 2). This collection insinuated superiority between the right gluteus minimus and medius muscles (Figure 2). There were destructive changes on both sides of the right SI joint, similar to the CT scan taken five months prior. Blood cultures were negative after aspiration at the time of the ED visit; then, the ED providers noted a differential diagnosis for sacroiliitis. She was given ketorolac (Toradol) for pain and discharged with an orthopedic outpatient follow-up.

**Figure 3:**
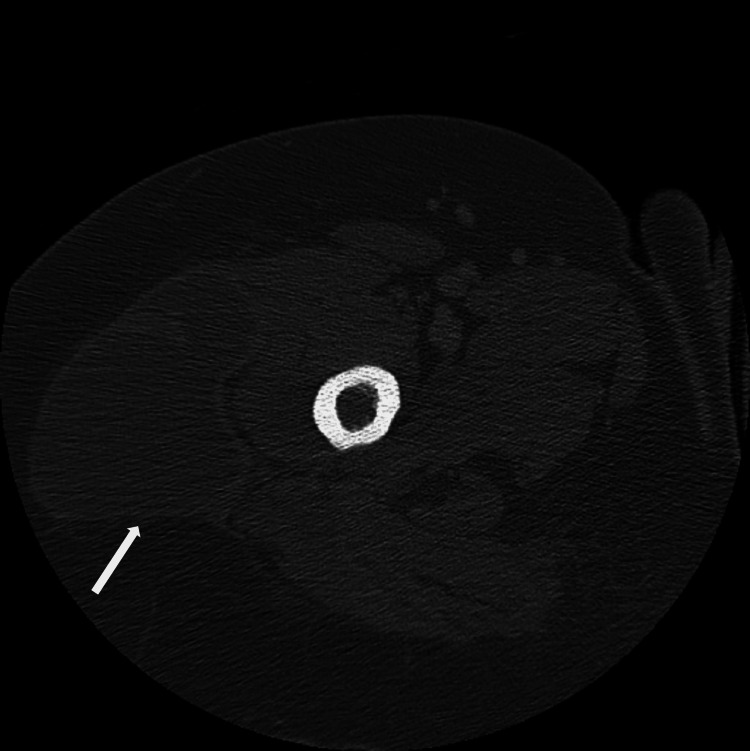
A CT of the right femur with contrast at a subsequent ED visit demonstrated large fluid collection findings similar to previous studies.

Over the course of the following month, the patient made outpatient visits to orthopedics, interventional radiology, and the ED. She endorsed a 40-pound weight loss over the past five months, further warranting a fluid aspiration and culture procedure in which three fluids were cultured, including two from the right hip and one from the right thigh. A CT of the right hip and femur with contrast taken at the subsequent ED visit approximately three weeks after the presentation showed a large fluid collection at the right hip region (Figure 3). Labs continued to show elevated ESR (66 mm/hr) and CRP (7.10 mg/dL).

Two weeks after the aspiration procedure, acid-fast bacilli cultures grew the Mycobacterium tuberculosis (MTB) complex without rifampin resistance. As such, the patient was seen by an outpatient infectious diseases specialist and was diagnosed with indolent extrapulmonary MTB. A contrast CT of the chest was obtained and revealed no communicable pulmonary MTB. Labs continued to be significant for elevated ESR (102 mm/hr) and CRP (2.78 mg/dL). The patient was not admitted to the hospital due to social issues and her inability to do well with new faces aside from her guardian. The patient was not started on MTB treatment right away due to concerns about drug-drug interactions with rifampin and rifabutin and the large number of pain and psychiatric medications that the patient was taking. These medications were prescribed by an outside psychiatry provider and included alprazolam, aripiprazole (Abilify), divalproex sodium (Depakote), fluphenazine (Prolixin), quetiapine (Seroquel), risperidone, sertraline (Zoloft), and trazodone. Psychiatry was consulted and was not concerned with toxicity; however, they noted the redundancy of medications at low, non-therapeutic doses and recommended their discontinuation. Ten days after the infectious disease visit, the patient was started on quadruple TB therapy of rifampin, isoniazid, pyrazinamide, and ethambutol; the patient was not started on pyridoxine. Per protocol, the patient was to be given intensive quadruple TB therapy for two months and assess the course (a total of six to nine months for treatment) at follow-up visits. The patient has been doing well clinically since this treatment.

## Discussion

ISI is an uncommon diagnosis, and its subsequent treatment course tends to be delayed and complicated for various reasons. Important risk factors for ISI include chronic diseases such as type II diabetes mellitus, immunosuppressive therapy, immunodeficiency diseases (HIV/AIDS), drug or alcohol addiction, cancer, and other infections [6]. Only 1% to 5% of infections of the musculoskeletal system are tuberculous in nature, and 3% to 9.7% of these cases involve the SI joint [7-9]. Due to the rarity of MTB infections of the SI joint and their non-specific clinical presentation as well as their mimicry of other diseases, this pathology is often left off the differential and leads to delays in diagnosis, as seen in this case. Despite their rarity, according to the 2022 Global Tuberculosis Report, MTB infections have risen 4.5% from 2020 to 2021 [10], and a concomitant rise in extrapulmonary manifestations was seen in previous years [11]. MTB infections pose a continuous, community-wide threat to individuals in developing nations [12]. A significant portion of MTB cases in countries with low incidence rates occurs among individuals emigrating from high-incidence areas [13]. Therefore, more robust and consistent screening processes are warranted. Many of the screening processes already in place involve proof of a plain chest radiograph [13], which may not have been as impactful for this extrapulmonary manifestation and potentially justify long-term follow-ups.

The complexity of this case lies not only in the two-year delay in diagnosis and the start of MTB treatment but also in the treatment management post-diagnosis. While navigation of care around the patient’s developmental delay and psychosis was crucial, additional barriers, such as outdated medication documentation, non-therapeutic usage of numerous psychiatric medications, and the need for psychiatry consultation, also led to delays in treatment. At the time of diagnosis, infectious diseases consultation had suggested that this process might have been expedited had the patient been hospitalized for a short stint of time. However, this was complicated by the patient’s lack of comfort around individuals other than her caregiver and her lack of social support to assist the caregiver in being present for the hospitalization. A pulmonary infection had yet to be ruled out following the MTB diagnosis. Per infection control policies, any patient who is suspected of having active infectious pulmonary tuberculosis should be placed in a TB isolation room with the appropriate ventilation facilities [11], which was not possible in this case due to complex social situations. Infectious disease specialists consulted the County TB Control Advisory Group for treatment recommendations before starting the patient on standard therapy. Vulnerability factors in patients with mental illness are often risk factors that predispose patients to MTB, such as poverty, homelessness, HIV, diabetes, and more [14]. A previous study on the prevalence of MTB infection in Ethiopian-specialized treatment centers found a high prevalence of MTB among patients with psychotic disorders, highlighting the need for more targeted interventions between these co-morbidities [14].

Previous case studies have explored rare extrapulmonary manifestations of MTB, including manifestations in the wrist joint and SI joint. They continuously emphasize the difficulty and importance of confirming a diagnosis early on to reduce long-term consequences and improve the odds of full recovery [15-19]. Classic anti-tubercular therapy (ATT) that targets microbial pathogenesis has been shown to be effective in treating MTB infections diagnosed earlier in their course. However, ATT has recently been under scrutiny for two reasons. One reason is the failure of ATT in the advent of increasing drug resistance [19]. Another reason is the necessity of having treatments that can help circumvent the barriers in place due to social determinants of health [20]. The latter of these two reasons has clearly been demonstrated in this case report. If the development of new treatment regimens against MTB can manage to address these two concerns, it will move the global community one step closer to equitable treatment of MTB cases and an eventual decrease in morbidity from this disease.

## Conclusions

Extrapulmonary manifestations of Mycobacterium tuberculosis are a rare type of tuberculosis infection in immunocompetent patients that occur outside of the usual respiratory system. In this report, a patient who emigrated from an endemic region was diagnosed with tuberculosis infection of the right sacroiliac joint. However, this presentation was further complicated due to the patient’s psychiatric and medication history, a delay in diagnosis or treatment, and socioeconomic factors. The steadfast recognition of this uncommon presentation alongside the development of equitable treatment regimens is a crucial next step in the world's fight against tuberculosis.
